# Increased roll tilt thresholds are associated with subclinical postural instability in asymptomatic adults aged 21 to 84 years

**DOI:** 10.3389/fnagi.2023.1207711

**Published:** 2023-08-10

**Authors:** Andrew R. Wagner, Megan J. Kobel, Daniel M. Merfeld

**Affiliations:** ^1^Department of Otolaryngology – Head and Neck Surgery, Ohio State University Wexner Medical Center, Columbus, OH, United States; ^2^School of Health and Rehabilitation Sciences, The Ohio State University, Columbus, OH, United States; ^3^Department of Speech and Hearing Science, The Ohio State University, Columbus, OH, United States; ^4^Department of Biomedical Engineering, The Ohio State University, Columbus, OH, United States

**Keywords:** vestibular, Aging, balance, postural control, vestibular threshold, perception

## Abstract

**Background:**

Balance assessments that intentionally alter the reliability of visual and proprioceptive feedback (e.g., standing on foam with eyes closed) have become a standard approach for identifying vestibular mediated balance dysfunction in older adults. However, such assessments cannot discern which specific element of the vestibular system (e.g., semicircular canal, otolith, or combined canal-otolith) underlies the observed age-related changes in balance performance. The present study was designed to determine the associations between specific sources of vestibular noise and quantitative measures of quiet stance postural control measured during standard “vestibular” balance conditions.

**Methods:**

A group of 52 asymptomatic adults (53.21 ± 19.7, 21 to 84 years) without a history of vestibular or neurologic disorders volunteered for this study. We measured a battery of five vestibular perceptual thresholds that assay vestibular noise with predominant contributions from the vertical canals, lateral canals, utricles, saccules, and the centrally integrated canal-otolith signal. In addition, participants completed two standard balance assessments that were each designed to prioritize the use of vestibular cues for quiet stance postural control—eyes closed on foam (Condition 4 of the Modified Romberg Balance Test) and eyes closed, on a sway referenced support surface (Condition 5 of the Sensory Organization Test).

**Results:**

In age adjusted models, we found strong positive associations between roll tilt vestibular thresholds, a measure of noise in the centrally integrated canal-otolith signal, and the root mean square distance (RMSD) of the anteroposterior and mediolateral center of pressure (CoP) captured during eyes closed stance on a sway referenced support surface. The strength of the association between roll tilt thresholds and the RMSD of the CoP was between 3-times and 30-times larger than the association between postural sway and each of the other vestibular thresholds measured.

**Conclusion:**

We posit that noise in the centrally estimated canal-otolith “tilt” signal may be the primary driver of the subclinical postural instability experienced by older adults during the “vestibular” conditions of balance assessments. Additional testing in adults with clinical balance impairment are needed to identify if roll tilt thresholds may also serve as a surrogate metric by which to detect vestibular mediated balance dysfunction and/or fall risk.

## Introduction

When standing on a solid surface in a well-lit room, sensory feedback from the visual system, lower extremities, and vestibular system each provide information used to minimize postural sway (Forbes et al., 2018). Distal proprioceptive cues from the lower extremities provide feedback about body sway relative to a reference frame defined by the surface upon which a person is standing, whereas visual cues are referenced to the visual environment. The vestibular system instead senses changes in head motion relative to a constant reference frame defined by gravity (Goldberg et al., 2012; Wolfe et al., 2021). As a result, balance tests can measure postural control under the presumed reliance upon the unperturbed vestibular inputs by modifying the testing environment to manipulate the veracity of visual (e.g., blindfold or moving visual scene) and support surface (e.g., standing on foam) cues. The observations of marked instability in patients with known vestibular lesions (Fetter et al., 1991) have made such assessment techniques a standard approach for identifying balance dysfunction mediated by impaired vestibular sensation (Nashner et al., 1982; Nashner and Peters, 1990; Horak, 2009; Wagner et al., 2021a), including in older adults.

In a large nationally representative sample of adults above 40 years of age, Agrawal and colleagues showed that the inability to stand on a foam pad (altering the reliability of proprioceptive cues) with eyes closed (removing visual cues) was associated with a significant increase in the likelihood of reporting a difficulty with falls (Agrawal et al., 2009). While these data strongly point to the vestibular system as being at least one of the primary contributors to age-related imbalance, balance assessments cannot isolate the influences of individual vestibular modalities on postural control. The vestibular system as a whole is often inextricably linked to balance, however, the peripheral vestibular system encapsulates ten individual sensors (three semicircular canals and two otolith organs in each ear) that collectively allow us to sense and respond to tilts, translations, and rotations of the head in the three-dimensions of space (Wolfe et al., 2021). Due to limited specificity of “vestibular” balance tests, the specific element of the vestibular sensory apparatus that leads to the observed age-related declines in balance is largely unknown.

Past attempts to define the specific contributions of the vestibular system to age-related imbalance have used clinical vestibular assays designed to probe the integrity of specific vestibular reflex pathways. However, the interpretation of correlations between these assessments and postural control measures are limited by (1) the reliance upon sensorimotor outcomes to indirectly infer sensory function and (2) fundamental differences in the methodologies used to probe function of the otoliths (e.g., vestibular evoked myogenic potentials) compared to the semicircular canals (vestibulo-ocular reflex) (see (Wagner et al., 2021a) for a review on this topic). The present study was designed to fill this gap by using a common experimental methodology—vestibular perceptual thresholds—to determine the relative associations between each aspect of the vestibular system (e.g., semicircular canals, otoliths, and the combined canal-otolith signal) and quiet stance postural control measured during traditional “vestibular” balance conditions.

Vestibular thresholds represent a behavioral assay of sensory noise (or conversely sensory precision) and are defined as the smallest motion stimulus that a person can reliably perceive when moved in a specific motion plane (e.g., rotation, tilt, or translation) known to preferentially excite a vestibular end organ (e.g., semicircular canals or otoliths) (Merfeld, 2011; Kobel et al., 2021b). Secondary to the closed loop nature of quiet stance postural sway (Peterka, 2002; Maurer and Peterka, 2005; Cenciarini and Peterka, 2006; van der Kooij and Peterka, 2011; van Kordelaar et al., 2018), we hypothesized that individuals with increased vestibular thresholds (i.e., greater sensory noise) would show greater variability (i.e., imprecision) in postural sway.

A previously published dataset in young adults showed a specific correlation between mid-frequency (i.e., 0.5 Hz) roll tilt vestibular thresholds and quiet stance postural sway during an eyes closed, on foam balance task (Wagner et al., 2021c). Similarly, roll tilt thresholds have been shown to correlate with the likelihood of being able to complete (i.e., stand for 30 s) the same eyes closed on foam balance task in a sample of adults over the age of 40 (Bermúdez Rey et al., 2016). Given these findings, alongside the presumed necessity to precisely estimate dynamic head in space orientation during quiet stance sway, we hypothesized that 0.5 Hz roll tilt vestibular thresholds—quantifying the precision in perceptual estimates of dynamic head-in-space orientation, and reflecting noise in the centrally integrated canal-otolith signal—would display the strongest correlation with quiet stance postural sway in conditions that remove vision and provide unreliable proprioceptive cues.

## Materials and methods

### Recruitment and study procedures

A total of fifty-four participants were recruited from The Ohio State University, as well as from surrounding regions of central Ohio. We recruited individuals within each of three age ranges: 18–39, 40–64, and 65–89 years of age. The delineation between young and middled aged adults was made based upon prior data showing an increase in vestibular thresholds beginning at age 40 (Bermúdez Rey et al., 2016). The designation of older adults as those participants ≥ 65 years of age was made based upon the definition adopted by the American Medical Association (Lundebjerg et al., 2017). Prior to enrollment, each participant completed questionnaires pertaining to their overall health and medical history. The responses provided were reviewed by a vestibular audiologist to identify the presence of any conditions that may impact balance or vestibular assessments. Participants were excluded if they reported having a neurologic disorder, vestibular disorder (excluding resolved BPPV), recent surgery, uncorrected visual impairment, diabetes, or a recent orthopedic injury (<6 months). In addition, due to mechanical constraints of the motion platform, a weight limit of 250 pounds was used for inclusion in the study. We also screened for the presence of frailty using the PRISMA-7; a score of < 3 was required for inclusion in the study (Dent et al., 2016). Given the attentional demands of perceptual threshold testing, as well as a previously identified association between cognitive impairment and balance (Semenov et al., 2016), we also screened for mild cognitive impairment (MCI) and possible undiagnosed dementia using the Self-Administered Gerocognitive Examination (SAGE) (Scharre et al., 2010). A cut off score of 16 or greater was used for inclusion in this study (Scharre et al., 2010).

Testing was broken up into 2 days (2–2.5 h each day) and each participant was compensated monetarily for their time spent in the lab. Threshold assessments and the instrumented Modified Romberg Balance Test (MRBT) were performed on the same day in a single session. We used a standard four condition MRBT protocol. The four MRBT conditions are illustrated in Supplementary Figure 1. To mitigate the effects of fatigue, the sensory organization test (SOT) was performed on a separate day. We used a standard six-condition SOT protocol. The six SOT conditions are also illustrated in Supplementary Figure 1. Additional tests of postural control were also collected during the second visit, and are reported in detail within the doctoral thesis of the lead author (Wagner, 2023). Within this published thesis, alternative analyses of the present data can also be found (Wagner, 2023). The study protocol was approved by the Ohio State University Institutional Review Board and each participant provided informed consent.

### Vestibular perceptual thresholds

Vestibular perceptual thresholds were measured using a direction recognition task, that we (Grabherr et al., 2008; Valko et al., 2012; Bermúdez Rey et al., 2016; Kobel et al., 2021a; Wagner et al., 2021c, 2022a,b), and others (MacNeilage et al., 2010; Crane, 2016; Keywan et al., 2020; Karmali et al., 2021) have used extensively to quantify vestibular precision. Motion stimuli were delivered using a MOOG (Aurora, NY) six degree of freedom (6DOF) motion platform. Participants were seated in a custom-built chair rigidly fixed to the motion platform. The head was restrained in a motorcycle helmet which was also rigidly mounted to the platform; this allowed for the motions of the head to be coupled to the platform, and for the participant to be moved en bloc (i.e., no motion at the cervical spine). All testing took place in a dark (light tight) room to remove the influence of visual motion cues. In addition, insert headphones were used to provide (1) passive sound attenuation (∼20 dB sound pressure level (SPL)) and (2) binaural white noise (at ∼60 dB SPL) during each test motion.

**FIGURE 1:**
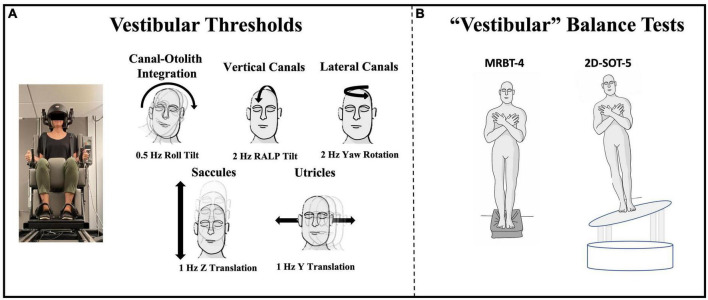
Vestibular threshold motions and the two quiet stance balance conditions focused upon herein are shown. **(A)** Each vestibular threshold was measured using a direction recognition task, with motion stimuli delivered using a MOOG 6DOF motion platform. Motion profiles of a specific trajectory and frequency were used to identify measures of noise with primary contributions from each vestibular end-organ or end-organ pair. Black arrows show the plane of motion for each threshold. **(B)** Center of pressure data were collected from force plates during condition 4 of the Modified Romberg Balance Test (MRBT-4; eyes closed, on foam) and a modified condition 5 of the Sensory Organization Test (2D-SOT-5; eyes closed, on a support surface sway referenced in both ML and AP planes) (Wagner and Merfeld, 2023).

For each threshold assessment, the participant was asked to indicate (using buttons in either hand) the perceived direction of motion (e.g., right vs. left). Each test consisted of 100 trials of a single motion profile (e.g., roll tilt) and the stimuli were adjusted using an adaptive 4-Down, 1-Up (4D1U) staircase procedure, with the step sizes determined using PEST rules (Leek, 2001). The motion stimulus used for each test was a single cycle of sinusoidal acceleration [a(t) = A sin (2π⨏t)] with the frequency of the motion being reflected by the inverse of the cycle duration (e.g., 2 Hz motion = 0.5 s per cycle). This yields a motion where the peak velocity [v = AT/π] and displacement [d = AT^2^/2π] are each proportional to acceleration (A) (Grabherr et al., 2008). In the present study, five distinct vestibular thresholds were measured (Figure 1):

(1) 1 Hz y-translation (sliding horizontally left or right) targeting the utricles (Fernandez and Goldberg, 1976; Valko et al., 2012; Kobel et al., 2021a).
(2) 1 Hz z-translation (sliding vertically up or down) targeting the saccules (Fernandez and Goldberg, 1976; Valko et al., 2012; Kobel et al., 2021a).
(3) 2 Hz RALP (right-anterior left-posterior) tilt (tilting the head forward and to the right or backward and to the left) targeting the vertical canals (Suzuki et al., 1964; Wagner et al., 2022a).
(4) 0.5 Hz roll tilt (tilting the head left or right in the coronal plane) targeting central canal-otolith integration (Angelaki et al., 2000, 2004; Angelaki and Yakusheva, 2009; Lim et al., 2017; Wagner et al., 2022a).
(5) 2 Hz yaw rotation (rotating in the horizontal plane about an earth vertical axis) targeting the lateral canals (Grabherr et al., 2008; Valko et al., 2012; Cousins et al., 2013; Priesol et al., 2014).

For each individual condition, the binary response data and stimulus magnitudes were fit to a Gaussian cumulative distribution function and the vestibular threshold parameter was estimated from a bias reduced generalized linear model (Chaudhuri et al., 2013). In the absence of bias, the “one-sigma” threshold parameter represents the stimulus magnitude that would be expected to, on average, yield an accuracy of 84.1%. A delete one jackknife approach was used to detect and remove attentional lapses during each threshold assessment (Clark and Merfeld, 2021).

### Quiet stance balance

Center of pressure (CoP) data were collected during two standard quiet stance balance assessments, the MRBT and SOT. The two primary sway measures were collected from the two conditions that each involved standing quietly with vision removed, and with the support surface cues made to be unreliable—MRBT-4 and 2D-SOT-5. Condition 4 of the modified Romberg balance test (MRBT-4) was performed with the eyes closed and while standing atop a medium density (5 lbs./ft3) foam pad of the dimensions 16” × 18” × 3’ (SunMate, Leicester, North Carolina). Stance was maintained for 67 s. During each trial, ambient auditory cues were mitigated using over the ear, active noise cancelling headphones (Bose Quiet Comfort II, Framingham, MA) with approximately 60 dB SPL of white noise added. Participants stood with their arms crossed at the chest and their feet positioned such that the medial borders of each foot were touching. CoP data were collected at 100 Hz from a triaxial AMTI force plate (Watertown, MA).

A modified condition 5 of the Sensory Organization Test (2D-SOT-5) was administered using a Virtualis MotionVR (Perols, Herault, France) motion platform. Participants stood with eyes closed and with the support surface sway referenced in both the mediolateral and anteroposterior directions (Wagner and Merfeld, 2023). CoP data were recorded at 90 Hz from two tri-axial force plates embedded within the platform. As in the MRBT-4, participants stood with their arms folded across the chest, with a narrow base of support, and were instructed to stand as still as possible. Active noise cancelling headphones were also worn throughout the SOT assessment. Consistent with the standard SOT procedures, three trials of 20 s each were completed, rather than a single trial of 67 s. In addition to the primary outcomes derived from performance on 2D-SOT-5 and MRBT-4, the remainder of the MRBT and SOT test conditions (Supplementary Figures 1, 2) were analyzed as secondary variables.

The raw CoP data from each balance assessment were processed off-line using a custom script written in Matlab (v2020a, Natick, MA). The CoP data were low pass filtered using a 4th order zero-phase-lag digital filter (filtfilt.m; MATLAB, Natick MA) with a 25 Hz cut off. The primary outcome measure, the root mean square distance (RMSD), was calculated by taking the standard deviation of the filtered and zero-meaned CoP signal for both the medio-lateral (ML) and anterior-posterior (AP) planes. To remove transient responses, the first 7 s of the MRBT trial was removed prior to the calculation of the RMSD and for the SOT, the median of the three trials was used.

### Statistical analysis

Vestibular perceptual thresholds have previously been shown to display a log normal distribution. Consistent with several past studies (Grabherr et al., 2008; Bermúdez Rey et al., 2016; Kobel et al., 2021a; Wagner et al., 2022a), each of the vestibular thresholds showed a lognormal distribution and were transformed prior to the analysis described below. Age effects were described using linear regression models with the regression coefficients extrapolated to estimate the percent change in thresholds per decade. Based on previous studies (Bermúdez Rey et al., 2016; Karmali et al., 2017; Beylergil et al., 2019; Wagner et al., 2021c), our *a priori* hypothesis was that roll tilt thresholds would positively correlate with sway (i.e., larger roll tilt thresholds would correlate with greater sway). Nonetheless, since none of the prior studies had quantified the relative strength of the associations between different thresholds and quantitative measures of postural sway, multivariable regression models were used to determine the association between each threshold measure and the RMSD of the CoP measured during MRBT-4 and 2D-SOT-5, while controlling for the effects of age. This was repeated for the both the ML and AP RMSD values. Postural sway data were only analyzed for those participants who were able to complete the individual test conditions without a loss of balance (*N* = 47 in MRBT-4 and *N* = 51 in 2D-SOT-5). Additionally, in five out of the twenty older adult participants, RALP tilt thresholds could not be collected due to the psychophysical staircase exceeding the displacement limits of the motion device. Since these data were not missing at random, we did not impute values to replace the missing data points. Instead, the association with RALP tilt thresholds was restricted to univariable regression analysis. The total sample size included in each analysis can be found within each table that follows.

Univariable linear regression models were used to characterize the association between each threshold measure and the RMSD of postural sway, both with and without adjusting for the effects of age. To account for the number of comparisons (twenty in total), and to also mitigate Type II errors inherent to Bonferroni correction (Perneger, 1998), the Benjamini-Hochberg False Discovery Rate method (FDR) was used to account for multiple comparisons (Benjamini and Hochberg, 1995). This method ranks the *p*-values from smallest to largest, and then sets a critical value based upon an acceptable level of error (0.05 or 5%), the rank of a given *p*-value (m), and total number of comparisons being made (*N*) (Critical Value = 0.05 * (m/*N*)) (Benjamini and Hochberg, 1995). Each *p*-value was compared to the critical value to determine statistical significance; the reported *p*-values are corrected using the same approach [*p* x (*N*/m)]. In addition to the primary analysis, separate age-adjusted univariable regression models were run to investigate the linear association between each threshold and the secondary sway outcomes (AP and ML RMSD) captured from the remaining conditions of the MRBT and SOT.

## Results

### Sample characteristics

We measured vestibular perceptual thresholds and quiet stance balance in a sample of 52 healthy adults between the ages of 21 and 84 (Mean = 53.21, SD = 19.7) (Table 1). Two of the 54 enrolled participants completed half of the test battery but did not return for the second session. The final sample (*N* = 52) included 17 young adults aged 18 to 39 (*N* = 17, Mean = 29.65, SD = 5.42, Range = 21 to 37), 15 middle aged adults aged 40 to 64 (*N* = 15, Mean = 52.07, SD = 6.02, Range = 44 to 64), and 20 older adults aged 65 and older (*N* = 20, Mean = 74.1, SD = 5.78, Range = 66 to 84). One older adult reported a remote history of BPPV but denied any current symptoms of positional vertigo; otherwise, no participants reported a history of vestibular or neurological disorders. Each individual lived independently in the community and ambulated into the research lab without use of an assistive device. In addition, none of the participants were found to be at risk for frailty based upon the PRISMA-7 (< 3) (Dent et al., 2016) and 51/52 of the participants scored above the cut off for MCI on the SAGE (Scharre et al., 2010) (Mean = 21.06, SD = 1.48, Range = 16 to 22); the total SAGE score for one participant was not available.

**TABLE 1:** Demographic data and descriptive statistics (Mean ± 1 SD) are shown for the primary variables of interest.

	Young adult	Middle aged	Older adult	Total
*N* (Female)	17 (9)	15 (11)	20 (13)	52 (33)
Age	29.65 ± 5.42	52.07 ± 6.02	74.1 ± 5.78	52.81 ± 20.20
ML MRBT-4 RMSD (mm)	10.74 ± 3.65	13.39 ± 4.13	15.36 ± 2.99	13.06 ± 4.04
AP MRBT-4 RMSD (mm)	11.14 ± 4.33	12.51 ± 3.54	14.46 ± 5.10	12.64 ± 4.49
ML 2D-SOT-5 RMD (mm)	23.03 ± 7.24	25.66 ± 3.46	27.18 ± 7.70	25.35 ± 6.68
AP 2D-SOT-5 RMD (mm)	15.54 ± 6.10	17.24 ± 3.31	17.79 ± 5.77	16.88 ± 5.28
Roll tilt threshold (°/s)	0.82 ± 0.27	1.11 ± 0.56	1.64 ± 0.96	1.22 ± 0.76
RALP tilt threshold (°/s)	0.63 ± 0.26	0.93 ± 0.33	1.91 ± 1.18*	1.13 ± 0.88
Yaw rotation threshold (°/s)	0.67 ± 0.30	1.21 ± 1.16	1.07 ± 0.55	0.98 ± 0.75
Z translation threshold (cm/s)	1.59 ± 0.88	2.88 ± 1.48	6.01 ± 3.03	3.66 ± 2.83
Y translation threshold (cm/s)	0.66 ± 0.40	0.78 ± 0.28	2.19 ± 2.33	1.28 ± 1.62

Due to falls, data were only available for *N* = 47 subjects for MRBT-4 and *N* = 51 subjects for 2D-SOT-5. In addition, RALP tilt thresholds could not be collected in 5 older adults due to the staircase surpassing the limits of the motion device. Threshold values are shown in original units, however data were log transformed prior to performing the statistical analysis. AP, anteroposterior; ML, mediolateral; RALP, right-anterior; left-posterior; MRBT, Modified Romberg Balance Test; RMSD, root mean square distance.

### Effect of age on vestibular thresholds

Age showed a significant positive linear relationship with each of the vestibular thresholds surveyed in this study, except for yaw rotation. Per decade, we found a 30.30% increase in the geometric mean of Z-translation thresholds (*p* < 0.0001), a 23.60% increase in Y translation thresholds (*p* < 0.0001), a 14.8% increase in roll tilt thresholds (*p* < 0.001), and a 22.9% increase in RALP tilt thresholds (*p* < 0.0001). For yaw rotation thresholds we saw only a trend toward a significant association with age (9.39% per decade, *p* = 0.09).

### Associations between vestibular thresholds and postural sway in 2D-SOT-5

**FIGURE 2:**
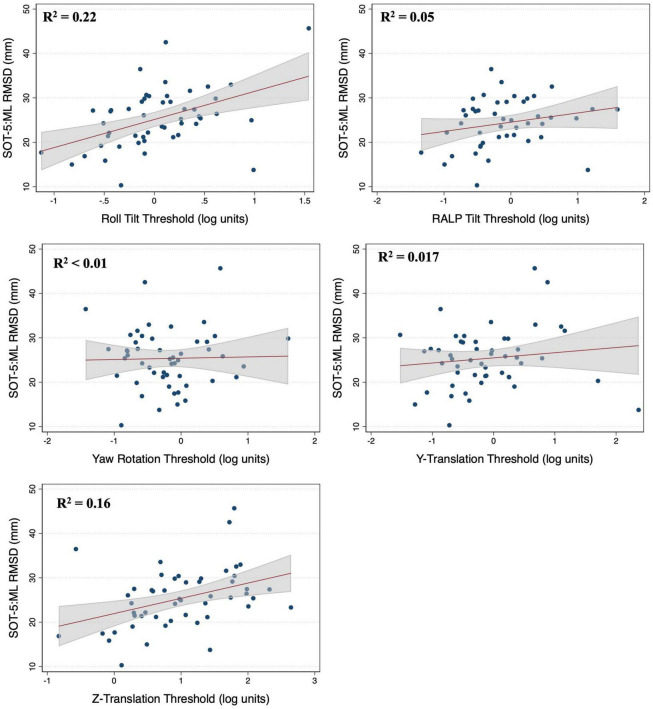
Scatter plots showing the association between each vestibular threshold and the mediolateral (ML) root mean square distance (RMSD) of the center of pressure in the “eyes closed, sway referenced support surface” condition (2D-SOT-5). A linear fit (red) and surrounding 95% confidence interval (gray) are shown. mm, millimeter.

**FIGURE 3:**
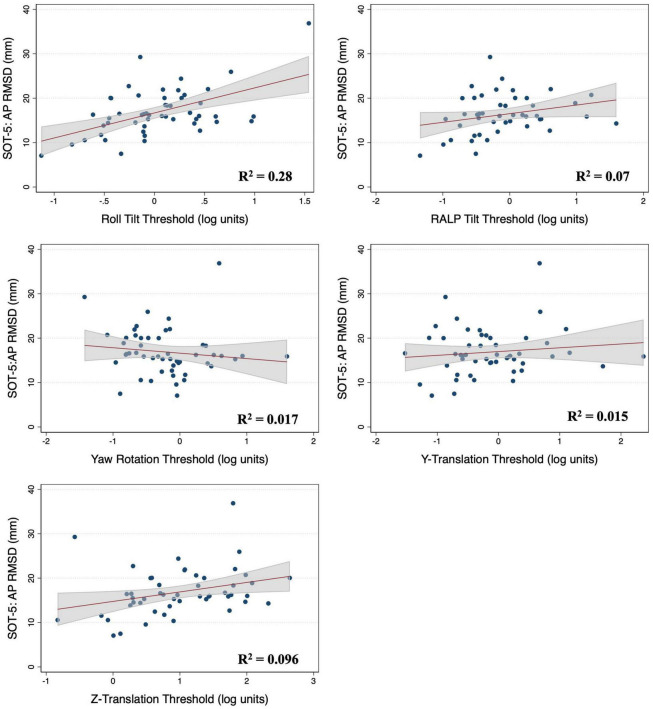
Scatter plots showing the association between each vestibular threshold and the anteroposterior (AP) root mean square distance (RMSD) of the center of pressure in the “eyes closed, sway referenced support surface” condition (2D-SOT-5). A linear fit (red) and surrounding 95% confidence interval (gray) are shown. mm, millimeter.

In multivariable regression models (*N* = 51), roll tilt perceptual thresholds showed significant positive associations with the ML (β = 6.77, *p* = 0.0093) and AP (β = 7.88, *p* < 0.0001) RMSD of the CoP when controlling for age and each of the remaining vestibular thresholds (Table 2). Yaw rotation thresholds also showed a weak, but significant, negative association with the AP RMSD (β = -2.64, *p* = 0.026). In each model, roll tilt thresholds showed the strongest association with the RMSD of the ML and AP RMSD (β_*stand*_ = 0.501 and 0.737 respectively) (Table 2). In the unadjusted univariable regression models (i.e., age excluded from each of these univariable models), roll tilt thresholds showed significant positive associations with the RMSD of ML (β = 6.33, *R*^2^ = 0.22, *p* = 0.0035) and AP (β = 5.64, *R*^2^ = 0.28, *p* = 0.001) CoP (Figures 2, 3). Z-translation (β = 3.43, *R*^2^ = 0.16, *p* = 0.021) thresholds also showed a significant positive association with the ML RMSD of the CoP in 2D-SOT-5. None of the remaining thresholds showed a significant association with either the ML or AP RMSD in the uncontrolled regression models (Table 3). After including age as a covariate in each model, only roll tilt thresholds continued to show a significant positive association with the ML (β = 5.90, *p* = 0.023) and AP RMSD (β = 6.34, *p* = 0.001) of the CoP.

**TABLE 2:** Results of multivariable linear regression models for test condition 2D-SOT-5 (*N* = 51).

	β	SE	*t*	*p*	β _stand_
**2D-SOT-5: Mediolateral CoP RMSD**					
**Roll tilt**	**6.77**	**2.49**	**2.72**	**0.0093**	**0.501**
**Yaw rotation**	**–1.50**	**1.57**	**–0.96**	**0.344**	**–0.128**
Y translation	–2.41	1.51	**–**1.59	0.118	**–**0.272
Z translation	1.07	1.87	0.57	0.571	0.123
Age	0.047	0.073	0.64	0.526	0.136
**2D-SOT-5: Anteroposterior CoP RMSD**					
**Roll tilt**	**7.88**	**1.81**	**4.35**	**<0.0001**	**0.737**
Yaw rotation	**–**2.64	1.14	**–**2.31	0.026	**–**0.284
Y translation	**–**1.99	1.10	**–**1.81	0.076	**–**0.284
Z translation	**–**0.20	1.36	**–**0.15	0.885	**–**0.029
Age	0.021	0.053	0.39	0.699	0.076

Standardized β values represent the amount of change in the response variable for a one standard deviation change in the predictor variable. Statistically significant (*p* < 0.05) variables are bolded. 2D-SOT-5 = A modified condition 5 of the Sensory Organization Test (eyes closed, on a support surface sway referenced in both ML and AP planes).

**TABLE 3:** Results of individual univariable linear regression models for test condition 2D-SOT-5.

2D-SOT-5	ML CoP RMSD			AP CoP RMSD			Sample size
	β	t-stat	*p*-value	β	t-stat	*p*-value	
**Roll tilt**	6.33 (5.90)	3.71 (2.89)	**0.0035 (0.023)**	5.64 (6.34)	4.34 (4.07)	**0.001(0.001)**	51
**RALP tilt**	2.08 (2.42)	1.57 (1.31)	0.225 (0.306)	1.94 (2.76)	1.82 (1.87)	0.167 (0.173)	47
**Yaw rotation**	0.002 (−0.007)	0.18 (−0.54)	0.907 (0.696)	−0.014 (−0.022)	−0.93 (−1.48)	0.509 (0.242)	51
**Y translation**	1.16 (−0.59)	0.92 (−0.39)	0.48 (0.777)	0.85 (0.089)	0.86 (0.07)	0.494 (0.943)	51
**Z translation**	3.43 (3.41)	3.00 (1.97)	**0.021** (0.154)	2.14 (2.58)	2.28 (1.82)	0.09 (0.15)	51

Model statistics for age-adjusted models are shown parenthetically. *P*-values were corrected using the False Discovery Rate (FDR) method. Significance was defined as a corrected *p*-value of <0.05 and is signified by the bolding of the significant *p*-values. 2D-SOT-5 = A modified condition 5 of the Sensory Organization Test (eyes closed, on a support surface sway referenced in both ML and AP planes). Sample size indicates the number of participants included in each analysis.

### Associations between vestibular thresholds and postural sway in MRBT-4

**FIGURE 4:**
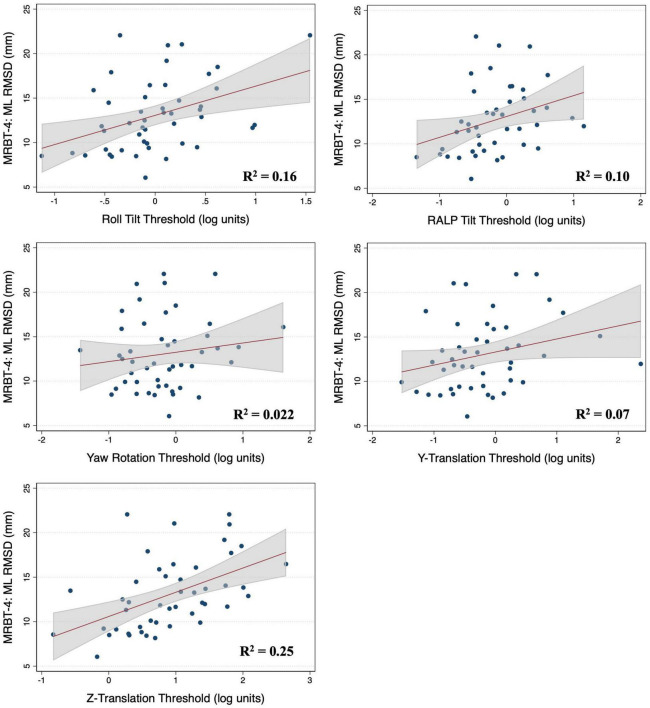
Scatter plots showing the association between each vestibular threshold and the mediolateral (ML) root mean square distance of the center of pressure in the “eyes closed, on foam” condition (MRBT-4). A linear fit (red) and surrounding 95% confidence interval (gray) are shown. mm, millimeter; RMSD, root mean square distance.

**FIGURE 5:**
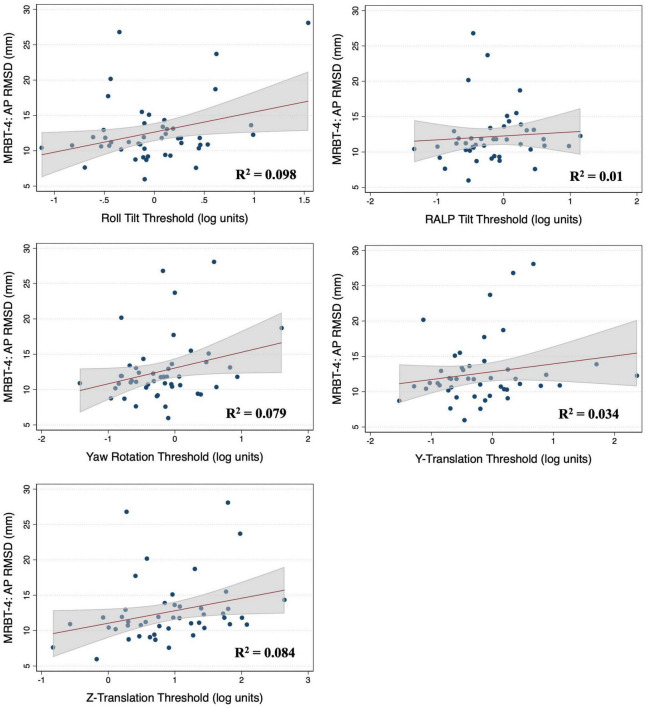
Scatter plots showing the association between each vestibular threshold and the anteroposterior (AP) root mean square distance (RMSD) of the center of pressure in the “eyes closed, on foam” condition (MRBT-4). A linear fit (red) and surrounding 95% confidence interval (gray) are shown. mm, millimeter.

In the multivariable analysis of the 47 adults who completed the MRBT-4 balance test, none of the individual thresholds showed a significant association with the RMSD of the ML or AP CoP (Table 4). Compared to the individual effects of each threshold, age showed the strongest association with ML and AP postural sway in the multivariable models (β_*stand*_ = 0.407 and 0.318 respectively) (Table 4). In the non-age adjusted univariable linear regression models, roll tilt (β = 3.28, *R*^2^ = 0.16, *p* = 0.040) and z-translation thresholds (β = 2.72, *R*^2^ = 0.25, *p* = 0.006) each showed significant positive associations with the ML RMSD of the CoP in the “eyes closed, on foam” condition (Figures 4, 5). For the AP RMSD no significant associations were found (Table 5). When age was included as a covariate, none of the five vestibular thresholds showed a significant association with either the ML or AP RMSD of the CoP (*p* > 0.05).

**TABLE 4:** Results of multivariable linear regression models for test condition MRBT-4 (*N* = 47).

	β	SE	*t*	*p*	β _stand_
**MRBT-4: Mediolateral CoP RMSD**					
Roll tilt	1.49	1.49	1.00	0.324	0.183
Yaw rotation	-0.735	1.01	-0.73	0.471	-0.103
Y translation	-0.640	0.92	-0.69	0.491	-0.118
Z translation	0.973	1.13	0.86	0.396	0.178
Age	0.086	0.044	1.97	0.056	0.407
**MRBT-4: Anteroposterior CoP RMSD**					
Roll tilt	2.01	1.83	1.10	0.277	0.222
Yaw rotation	1.054	1.24	0.85	0.399	0.133
Y translation	-0.686	1.13	-0.61	0.546	-0.114
Z translation	-0.418	1.39	-0.30	0.765	-0.069
Age	0.075	0.054	1.40	0.169	0.318

Standardized β values represent the amount of change in the response variable for a one standard deviation change in the predictor variable. Results significant at *p* < 0.05. MRBT-4 = condition 4 of the Modified Romberg Balance Test (eyes closed, on a foam surface).

**TABLE 5:** Results of individual linear regression models for test condition MRBT-4.

MRBT-4	ML CoP RMSD			AP CoP RMSD			Sample size
	β	t-stat	*p*-value	β	t-stat	*p*-value	
Roll tilt	3.28 (1.59)	2.96 (1.19)	**0.040** (0.378)	2.84 (1.56)	2.21 (1.08)	0.171 (0.415)	47
RALP tilt	2.35 (0.295)	2.21 (0.22)	0.128 (0.946)	0.55 (−1.79)	0.48 (−1.23)	0.843 (0.360)	47
Yaw rotation	0.021 (−0.011)	1.00 (−0.50)	0.325 (0.619)	0.036 (0.020)	1.97 (1.06)	0.055 (0.295)	47
Y translation	1.47 (−0.166)	1.89 (−0.20)	0.173 (0.845)	1.11 (−0.219)	1.26 (−0.21)	0.380 (0.886)	47
Z translation	2.72 (1.45)	3.85 (1.49)	**0.006** (0.329)	1.77 (0.35)	2.03 (0.29)	0.154 (0.949)	47

Model statistics for the age-adjusted models are shown parenthetically. *P*-values were corrected using the False Discovery Rate (FDR) method. Significance was defined as a corrected *p*-value of <0.05 and is signified by the bolding of the significant *p*-values. MRBT-4 = condition 4 of the Modified Romberg Balance Test (eyes closed, on a foam surface). Sample size indicates the number of participants included in each analysis.

### Associations between vestibular thresholds and sway during alternative balance conditions

In the secondary analysis of the CoP data captured from conditions 1-3 of the MRBT (Supplementary Figure 1) and conditions 1-4 and 6 of the SOT (Supplementary Figure 2), none of the threshold measures showed significant associations with the CoP RMSD in age adjusted regression models. Consistent with the analysis of the primary outcome variables, the strongest associations observed were between roll tilt thresholds and postural sway, and, to a lesser extent, z-translation thresholds and postural sway (Supplementary Figure 3).

## Discussion

In support of our primary hypothesis, we showed that 0.5 Hz roll tilt perceptual thresholds, reflecting noise in the centrally integrated canal-otolith roll tilt signal, displayed the strongest association with quiet stance postural control in the “vestibular” balance conditions that removed visual feedback and provided unreliable proprioceptive feedback (MRBT-4 and 2D-SOT-5). The specific association between roll tilt perceptual thresholds and the RMSD of the CoP signal, a measure of sway variability, suggests that imprecision (or noise) in the dynamic estimation of head in space orientation is associated with increased noise in the sensorimotor output of the postural control system. Below we discuss these findings in the context of the available literature, as well as provide a putative mechanistic explanation for the identified link between roll tilt perceptual thresholds and quiet stance postural sway.

### Comparison to past findings

Our findings are consistent with previously published data comparing perceptual thresholds to a categorical measure of “pass/fail” balance in condition 4 of the MRBT (i.e., eyes closed, on foam). Bermudez-Rey and colleagues showed that 0.2 Hz roll tilt thresholds (1) were a strong predictor of the ability to complete (i.e., stand 30 s) the “eyes closed, on foam” balance task (Bermúdez Rey et al., 2016; Karmali et al., 2017) and (2) mediated 46% of the age effect on balance (Beylergil et al., 2019). In a recent study of young adults (*N* = 33; 21 to 32 years of age), we also showed a significant positive correlation between 0.5 Hz roll tilt thresholds and the ML RMSD of the CoP captured during this same balance task (Wagner et al., 2021c). However, to this point, quantitative measures of postural sway had yet to be compared to roll tilt vestibular thresholds in a sample that included adults over the age of 40. The present study fills a gap left by these earlier studies, by showing that 0.5 Hz roll tilt thresholds display a significant positive association with quantitative measures of quiet stance postural sway, as measured using two independent assessments of postural control (MRBT-4 and 2D-SOT-5), in a sample of adults with a broad age distribution (21 to 84 years of age).

Karmali et al. (2021) also recently compared y-translation, z-translation, yaw rotation, and roll tilt (1 and 0.2 Hz) thresholds to the ML and the AP RMSD of the CoP during the SOT (Karmali et al., 2021). In a secondary experiment, several 0.2 Hz tilt thresholds (roll, pitch, left-anterior right posterior (LARP) and RALP) were also compared to postural sway during each condition of the MRBT. The only significant correlation found across both arms of the study was between 1 Hz y-translation thresholds and the ML RMSD in 2D-SOT-5 (i.e., eyes closed, sway referenced support) (Karmali et al., 2021). In the present study, 1 Hz y-translation thresholds showed minimal association with postural sway in either 2D-SOT-5 or MRBT-4. Although surprising, differences in the study populations likely explains this incongruous finding. Karmali and colleagues analyzed a sample of twelve, young to middle aged adults (mean age of 34 ± 9, range of 21–50), whereas our dataset included fifty-two adults between the ages of 21 and 84. The effect of y-translation thresholds shown by Karmali et al. (2021) may have therefore represented a finding specific to the young adult population or may have been simply a result of sampling variability. In support of the explanation based upon sampling, we failed to show a significant correlation between y-translation thresholds and the ML RMSD in 2D-SOT-5 when our analysis was repeated using only the 17 participants under the age of 40 (*r* = 0.04, *p* = 0.88).

Karmali et al. (2021) also failed to identify a significant association between roll tilt thresholds and measures of postural sway. Yet, while similar, our two studies differed in the frequency of the roll tilt stimulus used—Karmali, et al. used a 0.2 Hz motion, compared to 0.5 Hz in the present study. Recently we showed that 0.2 Hz (in addition to 1 Hz) roll tilt thresholds did not show a significant correlation with the RMSD of the ML CoP in an identical “eyes closed, on foam” balance task in a sample of young adults (Wagner et al., 2021c). Yet, as described above, in a study that instead sampled across a wider age distribution (18 to 80 years old), Bermudez-Rey and colleagues showed that an increase in 0.2 Hz roll tilt thresholds was a strong predictor of the likelihood of completing the “eyes closed, on foam” balance task (Bermúdez Rey et al., 2016). In a recent study of 37 healthy adults, Gabriel and colleagues also showed that pitch tilt perception was significantly correlated with the total CoP path length during a similar “eyes closed, on foam” quiet stance balance task, but only in the subset of adults over the age of 65 (*N* = 19) (Gabriel et al., 2022). Collectively these findings suggest that the absence of a significant correlation between 0.2 Hz roll tilt thresholds and quantitative measures of quiet stance sway (Karmali et al., 2021; Wagner et al., 2021c) likely resulted from the inclusion of only young healthy adult participants in these previous studies. Since neither Bermúdez Rey et al. (2016), nor the present study, included both 0.2 and 0.5 Hz roll tilt thresholds, we cannot fully discern which, if either, is a superior metric for quantifying the influence of noisy canal-otolith integration on age-related imbalance. However, since (a) the processing of 0.2 and 0.5 Hz roll tilt cues each requires the dynamic integration of canal and otolith signals (Lim et al., 2017; Wagner et al., 2022a), (b) each measure provides relevant cues for head in space orientation, and (c) 0.2 and 0.5 Hz roll tilt thresholds have been shown to be strongly correlated with one another (Lim et al., 2017; Wagner et al., 2021c), we expect that such differences would be small. Since multiple studies (Bermúdez Rey et al., 2016; Karmali et al., 2017; Beylergil et al., 2019; Wagner et al., 2021c) suggest that increased roll tilt thresholds show a robust association with subclinical balance dysfunction, optimizing the roll tilt test frequency may prove beneficial.

### Proposed mechanistic link between roll tilt vestibular noise and postural sway

A roll tilt of the head to the right, and a linear acceleration of the head to the left, each cause the hair cells embedded within the neuroepithelium of the utricles to deflect in an identical fashion (Baloh et al., 2011). As a result of this ambiguity in the otolith signal, during roll tilt, signals from the otolith organs alone cannot differentiate between changes in gravitoinertial force (the combined acceleration from translation and gravity) that occur secondary to (1) the head being tilted *versus* (2) the head being translated horizontally (Angelaki et al., 1999). As a result, to achieve a precise estimate of the head’s orientation relative to gravity, the brain must use an internal model to combine angular velocity signals from the vertical semicircular canals with the ambiguous gravitoinertial forces encoded by the otolith organs (Angelaki et al., 1999; Lim et al., 2017; Wagner et al., 2022a). Considering the inverted pendulum dynamics of quiet stance sway, the ability to dynamically sense the orientation of the head in space holds a clear ecological advantage for the maintenance of stable quiet stance balance.

In quiet stance, corrective torques from the distal lower extremities are generated directly in response to the sense of the body’s deviation away from upright (Peterka, 2002; Maurer and Peterka, 2005; Cenciarini and Peterka, 2006; van der Kooij and Peterka, 2011; Assländer and Peterka, 2014; Pasma et al., 2015; Peterka et al., 2017). Secondary to the closed loop nature of the system, imprecision in dynamic estimates of head orientation (resulting from noise in the vestibular tilt signal) would therefore be expected to yield an increase in the variability and/or amplitude of postural sway (van der Kooij and Peterka, 2011; Diaz-Artiles and Karmali, 2021); furthermore, the effects should be greatest in conditions where vestibular inputs are prioritized due to the removal of visual cues and the degradation of proprioceptive cues. By using an empirical measure of noise in the centrally derived estimate of head in space orientation—0.5 Hz roll tilt thresholds—here we showed that individuals with increased vestibular noise demonstrated greater variability (i.e., greater RMSD) in CoP displacement during two balance conditions that each degrade the veracity of non-vestibular sensory feedback (i.e., 2D-SOT-5 and MRBT-4). The agreement between our empirical findings, and the anticipated effects on sway that should, according to theory, result from an increase in vestibular noise, support our suggestion that when exposed to impoverished non-vestibular sensory cues, older adults show greater postural sway primarily due to greater noise in the vestibular tilt signal.

However, although our data are supportive of the hypothesized association between roll tilt thresholds and subclinical postural instability, due to our cross-sectional design, we cannot determine if roll tilt vestibular noise was the cause of the observed increase in RMS sway. Instead, it remains possible that postural control and vestibular precision change in parallel with age, without causal interactions. However, while acknowledging that correlation cannot prove causation, we posit that the available data points to vestibular noise being at least one of the primary contributors to subclinical postural instability (see Wagner et al., 2021b for a mini review we penned on this topic). This supposition of a causal link between vestibular noise and postural sway is based on several factors, including (1) our data showing that roll tilt thresholds displayed a significant positive association with postural sway in age adjusted models, across a broad distribution of adults ranging in age between 21 and 84 years, (2) our finding that although roll tilt thresholds showed the strongest association with postural sway, they displayed one of the weakest associations with age, and (3) previously published data showing that a paradigm designed to improve roll tilt perception was able to yield a significant reduction in sway (Wagner et al., 2022b). In addition to these empirical findings, roll tilt represents an ecologically valid signal to encode human postural sway given the inverted pendulum dynamics of the human body. For these reasons, we posit that the observed relationship between vestibular noise and postural sway is likely causal such that age-related increases in roll tilt vestibular noise contribute to subclinical postural instability in asymptomatic adults.

### Vestibular contributions to sway on a “foam” vs a “sway referenced” support surface

The results of the simple linear regression analysis showed that roll tilt thresholds explained a greater amount of the variance in postural sway in the two-dimensional “sway referenced” (2D-SOT-5), compared to the “foam standing” (MRBT-4) balance condition (ML: *R*^2^ = 0.22 vs. 0.16 and AP: *R*^2^ = 0.28 vs. 0.098). In the multivariable analysis of sway in the “eyes closed, on foam” condition, the effect of age was greater than that of roll tilt thresholds (Tables 2, 3), and in the individual age-adjusted linear regression models, the effect of age either remained significant (ML RMSD, *p* = 0.006), or trended toward significance (AP RMSD, *p* = 0.083), when controlling for roll tilt thresholds (Supplementary Table 1). Conversely, in the multivariable analysis of 2D-SOT-5 performance, the associations between roll tilt thresholds and RMS sway were stronger than the effects of age (as quantified by the standardized β, Table 2) and in the age-adjusted individual regression models, age did not show a significant effect on the ML (*p* = 0.693) or AP RMSD (0.374) when controlling for 0.5 Hz roll tilt thresholds (Supplementary Table 1).

These data suggest that the variation in postural sway in the “eyes closed, on foam” condition may primarily be explained by the variance in alternative age-related sensorimotor factors (e.g., tactile sensation, strength) (Ko et al., 2015; Deshpande et al., 2016), whereas postural sway in the “eyes closed, sway referenced support” condition appears instead to be more strongly influenced by roll tilt vestibular noise. This is further supported by an increase in the coefficients of determination in the linear regression models relating age to postural sway in MRBT-4 (AP: *R*^2^ = 0.14, ML: *R*^2^ = 0.27), compared to the 2D-SOT-5 (AP: *R*^2^ = 0.037, ML: *R*^2^ = 0.087). Future studies should test this supposition by comparing the postural responses during each of these assessments in a sample of adults with known vestibular lesions.

### Clinical relevance and applications

As measures of postural control have been shown to be insensitive to vestibular lesions (Nashner and Peters, 1990; Voorhees, 1990; Di Fabio, 1995), increased sway in the “vestibular” condition of a balance assessment cannot be reliably used as a marker of vestibular pathology in older adults (Evans and Krebs, 1999; Jacobson et al., 2011). Based upon the present data, we suggest that a concordant finding of both imbalance (e.g., increased sway in MRBT-4 or 2D-SOT-5), alongside an elevation in 0.5 Hz roll tilt thresholds, may serve as potential evidence of a vestibular mediated balance syndrome. However, the ability to generalize these findings to symptomatic older adults with clinical balance impairment and a falls history is limited by the healthy nature of our sample. In addition, our data also point to roll tilt precision as a potential target for future interventions that aim to improve postural control in older adults (Wagner et al., 2022b).

### Limitations

Increased sway variability, as reflected by the CoP RMSD, does not definitively connote worse postural control. Others have posited that changes in quiet stance balance may reflect greater exploratory behavior, rather than an unstable postural control system (Carpenter et al., 2010). Thus, the relationships between perceptual thresholds and either the velocity of sway or the frequency of sway may differ from those shown here for the RMSD. Future investigations may benefit from the inclusion of such measures, as well as alternative non-linear computational approaches (e.g., sample entropy) to further characterize the associations between vestibular noise and quiet stance balance. Additionally, although alternative “non-vestibular” influences (e.g., attention, tactile cues, etc.) are unavoidable during the assessment of vestibular thresholds, such factors similarly influence each of the thresholds measured, and thus do not prevent the use of vestibular perceptual thresholds to infer the relative contributions of each vestibular modality to age-related changes in quiet stance postural control. At the stimulus frequencies tested here (0.5 to 2 Hz) vestibular perceptual thresholds were also previously found to be 2.03-56.78 times higher in patients with absent bilateral vestibular function (due to bilateral labyrinthectomy), further supporting the predominant use of vestibular cues when perceiving passive whole body self-motion cues in the dark (Valko et al., 2012).

### Conclusion

Our data show a link between vestibular noise, specifically associated with the processing of roll tilt self-motion cues, and the variability of postural sway during quiet stance. These data support that noise in the centrally integrated canal-otolith signal, relative to the canal or otolith signals in isolation, may be the primary vestibular contributor to quiet stance postural sway in conditions of unreliable visual and proprioceptive cues. Consistent with our primary hypothesis, this suggests that imprecision in the dynamic estimation of head in space orientation, as represented by increased 0.5 Hz roll tilt perceptual thresholds, may contribute to subclinical postural instability observed in asymptomatic older adults.

## Data availability statement

The raw data supporting the conclusions of this article will be made available by the authors, without undue reservation.

## Ethics statement

The studies involving human participants were reviewed and approved by The Ohio State University Biomedical Sciences Institutional Review Board. The patients/participants provided their written informed consent to participate in this study.

## Author contributions

AW, MK, and DM conceptualized the experiments. AW and MK collected and processed the behavioral data. AW performed the statistical analyses, interpreted the results of the analyses, and wrote the initial draft of the manuscript. DM and MK each contributed to, and approved of, the final version of the manuscript. All authors contributed to the article and approved the submitted version.

## Supplementary material

Supplementary Figure 1 Alternative conditions of the Modified Romberg Balance Test (MRBT) are shown. MRBT-1 involved standing on a firm surface with eyes open, MRBT-2 involved standing on a firm surface with eyes closed. MRBT-3 and MRBT-4 were performed on a compliant medium density foam pad with eyes open in MRBT-3 and closed in MRBT-4. Each condition lasted 67 s and noise cancelling headphones were worn throughout. Center of pressure data were collected at 100 Hz from a tri-axial force plate.

Supplementary Figure 2 Alternative conditions of the Sensory Organization Test (SOT) are shown. In SOT-1, SOT-2, and SOT-3 the platform was stationary whereas during 2D-SOT-4, 2D-SOT-5 (Figure 1), and 2D-SOT-6, the platform tilted in the sway referenced conditions in two dimensions (anteroposterior and mediolateral) in response to an estimate of the displacement of the center of gravity. In SOT-1 and SOT-3 the eyes were open, and the participant viewed a veridical visual scene, in SOT-2 and 2D-SOT-5 the eyes were closed, and in SOT-3 and 2D-SOT-6 the VR goggles provided a sway referenced visual scene. The black masks in SOT-3 and 2D-SOT-6 denote the use of VR to provide a sway referenced visual scene. While VR goggles were worn throughout, they are removed from the graphic in the remaining tasks to allow visualization of the eyes (open vs. closed).

Supplementary Figure 3 Pearson correlation coefficients are provided within each box to demonstrate the strength of association between each of the secondary conditions of the sensory organization test (SOT conditions 1-4 and 6) and Modified Romberg Balance Test (MRBT conditions 1-3) and each of the five vestibular threshold measures—0.5 Hz roll tilt, 2 Hz RALP tilt, 2 Hz yaw rotation, 1 Hz Y-Translation, and 1 Z-Translation. The strength of the correlation is graphically indicated by the color of each cell, ranging from dark red (strongest positive association) to dark blue (strongest negative association).

## References

[B1] Agrawal Y. Carey J. Della Santina C. Schubert M. Minor L. (2009). Disorders of balance and vestibular function in US adults: Data from the National Health and Nutrition Examination Survey, 2001-2004. *Arch. Intern. Med.* 169 938–944. 10.1001/archinternmed.2009.66 19468085

[B2] Angelaki D. Yakusheva T. (2009). How vestibular neurons solve the Tilt/Translation Ambiguity: Comparison of brainstem, cerebellum, and thalamus. *Ann. N. Y. Acad. Sci.* 1164 19–28. 10.1111/j.1749-6632.2009.03939.x 19645876 PMC2860452

[B3] Angelaki D. McHenry M. Dickman J. Newlands S. Hess B. (1999). Computation of inertial motion: Neural strategies to resolve ambiguous otolith information. *J. Neurosci.* 19 316–327. 10.1523/JNEUROSCI.19-01-00316.1999 9870961 PMC6782388

[B4] Angelaki D. Merfeld D. Hess B. (2000). Low-frequency otolith and semicircular canal interactions after canal inactivation. *Exp. Brain Res.* 132 539–549. 10.1007/s002210000364 10912835

[B5] Angelaki D. Shaikh A. Green A. Dickman J. (2004). Neurons compute internal models of the physical laws of motion. *Nature* 430 560–564. 10.1038/nature02754 15282606

[B6] Assländer L. Peterka R. (2014). Sensory reweighting dynamics in human postural control. *J. Neurophysiol.* 111 1852–1864. 10.1152/jn.00669.2013 24501263 PMC4044370

[B7] Baloh M. Faan R. Honrubia M. DMSc V. Kerber M. K. (2011). *Baloh and Honrubia’s clinical neurophysiology of the vestibular system, fourth edition.* Oxford: Oxford University Press.

[B8] Benjamini Y. Hochberg Y. (1995). Controlling the false discovery rate: A practical and powerful approach to multiple testing. *JR Statist. Soc.* B, 289–300. 10.1111/j.2517-6161.1995.tb02031.x

[B9] Bermúdez Rey M. Clark T. Wang W. Leeder T. Bian Y. Merfeld D. (2016). Vestibular perceptual thresholds increase above the age of 40. *Front. Neurol.* 7:162. 10.3389/fneur.2016.00162 27752252 PMC5046616

[B10] Beylergil S. Karmali F. Wang W. Bermúdez Rey M. Merfeld D. (2019). Vestibular roll tilt thresholds partially mediate age-related effects on balance. *Prog. Brain Res.* 248 249–267. 10.1016/bs.pbr.2019.04.019 31239136

[B11] Carpenter M. Murnaghan C. Inglis J. (2010). Shifting the balance: Evidence of an exploratory role for postural sway. *Neuroscience* 171 196–204. 10.1016/j.neuroscience.2010.08.030 20800663

[B12] Cenciarini M. Peterka R. (2006). Stimulus-dependent changes in the vestibular contribution to human postural control. *J. Neurophysiol.* 95 2733–2750. 10.1152/jn.00856.2004 16467429

[B13] Chaudhuri S. Karmali F. Merfeld D. (2013). Whole body motion-detection tasks can yield much lower thresholds than direction-recognition tasks: Implications for the role of vibration. *J. Neurophysiol.* 110 2764–2772. 10.1152/jn.00091.2013 24068754 PMC3882814

[B14] Clark T. Merfeld D. (2021). Statistical approaches to identifying lapses in psychometric response data. *Psychon. Bull. Rev.* 28 1433–1457. 10.3758/s13423-021-01876-2 33825094

[B15] Cousins S. Kaski D. Cutfield N. Seemungal B. Golding J. Gresty M. (2013). Vestibular perception following acute unilateral vestibular lesions. *PLoS One* 8:e61862. 10.1371/journal.pone.0061862 23671577 PMC3650015

[B16] Crane B. (2016). Perception of combined translation and rotation in the horizontal plane in humans. *J. Neurophysiol.* 116 1275–1285. 10.1152/jn.00322.2016 27334952 PMC5023413

[B17] Dent E. Kowal P. Hoogendijk E. (2016). Frailty measurement in research and clinical practice: A review. *Eur. J. Intern. Med.* 31 3–10. 10.1016/j.ejim.2016.03.007 27039014

[B18] Deshpande N. Simonsick E. Metter E. Ko S. Ferrucci L. Studenski S. (2016). Ankle proprioceptive acuity is associated with objective as well as self-report measures of balance, mobility, and physical function. *Age* 38:53. 10.1007/s11357-016-9918-x 27146830 PMC5005915

[B19] Di Fabio R. (1995). Sensitivity and specificity of platform posturography for identifying patients with vestibular dysfunction. *Phys. Therapy* 75 290–305. 10.1093/ptj/75.4.290 7899487

[B20] Diaz-Artiles A. Karmali F. (2021). Vestibular precision at the level of perception, eye movements, posture, and neurons. *Neuroscience* 468 282–320. 10.1016/j.neuroscience.2021.05.028 34087393 PMC9188304

[B21] Evans M. Krebs D. (1999). Posturography does not test vestibulospinal function. *Otolaryngol. Head Neck Surg.* 120 164–173. 10.1016/S0194-5998(99)70401-8 9949347

[B22] Fernandez C. Goldberg J. (1976). Physiology of peripheral neurons innervating otolith organs of the squirrel monkey. II. Directional selectivity and force-response relations. *J. Neurophysiol.* 39 985–995. 10.1152/jn.1976.39.5.985 824413

[B23] Fetter M. Diener H. Dichgans J. (1991). Recovery of postural control after an acute unilateral vestibular lesion in humans. *J. Vestib. Res.* 1 373–383. 10.3233/VES-1991-1405 1670169

[B24] Forbes P. Chen A. Blouin J. (2018). Sensorimotor control of standing balance. *Handb. Clin. Neurol.* 159 61–83. 10.1016/B978-0-444-63916-5.00004-5 30482333

[B25] Gabriel G. Harris L. Gnanasegaram J. Cushing S. Gordon K. Haycock B. (2022). Age-related changes to vestibular heave and pitch perception and associations with postural control. *Sci. Rep.* 12:6426. 10.1038/s41598-022-09807-4 35440744 PMC9018785

[B26] Goldberg J. Wilson V. Cullen K. Angelaki D. Broussard D. Buttner-Ennever J. (2012). *The vestibular system: A sixth sense [Internet].* Oxford: Oxford University Press.

[B27] Grabherr L. Nicoucar K. Mast F. Merfeld D. (2008). Vestibular thresholds for yaw rotation about an earth-vertical axis as a function of frequency. *Exp. Brain Res.* 186 677–681. 10.1007/s00221-008-1350-8 18350283

[B28] Horak F. (2009). Postural compensation for vestibular loss. *Ann. N. Y. Acad. Sci.* 1164 76–81. 10.1111/j.1749-6632.2008.03708.x 19645883 PMC3224857

[B29] Jacobson G. McCaslin D. Piker E. Gruenwald J. Grantham S. Tegel L. (2011). Insensitivity of the “Romberg test of standing balance on firm and compliant support surfaces” to the results of caloric and VEMP tests. *Ear Hear.* 32 e1–e5. 10.1097/AUD.0b013e31822802bb 21775891

[B30] Karmali F. Bermúdez Rey M. Clark T. Wang W. Merfeld D. (2017). Multivariate analyses of balance test performance, vestibular thresholds, and age. *Front. Neurol.* 8:578. 10.3389/fneur.2017.00578 29167656 PMC5682300

[B31] Karmali F. Goodworth A. Valko Y. Leeder T. Peterka R. Merfeld D. (2021). The role of vestibular cues in postural sway. *J. Neurophysiol.* 125 672–686. 10.1152/jn.00168.2020 33502934 PMC7948142

[B32] Keywan A. Dietrich H. Wuehr M. (2020). Subliminal passive motion stimulation improves vestibular perception. *Neuroscience* 441 1–7. 10.1016/j.neuroscience.2020.05.053 32505748

[B33] Ko S. Simonsick E. Deshpande N. Ferrucci L. (2015). Sex-specific age associations of ankle proprioception test performance in older adults: Results from the Baltimore Longitudinal Study of Aging. *Age Ageing* 44 485–490. 10.1093/ageing/afv005 25637144 PMC4411223

[B34] Kobel M. Wagner A. Merfeld D. (2021a). Impact of gravity on the perception of linear motion. *J. Neurophysiol.* 126 875–887. 10.1152/jn.00274.2021 34320866 PMC8461827

[B35] Kobel M. Wagner A. Merfeld D. Mattingly J. (2021b). Vestibular thresholds: A review of advances and challenges in clinical applications. *Front. Neurol.* 12:643634. 10.3389/fneur.2021.643634 33679594 PMC7933227

[B36] Leek M. (2001). Adaptive procedures in psychophysical research. *Percept. Psychophys.* 63 1279–1292. 10.3758/BF03194543 11800457

[B37] Lim K. Karmali F. Nicoucar K. Merfeld D. (2017). Perceptual precision of passive body tilt is consistent with statistically optimal cue integration. *J. Neurophysiol.* 117 2037–2052. 10.1152/jn.00073.2016 28179477 PMC5434481

[B38] Lundebjerg N. Trucil D. Hammond E. Applegate W. (2017). When it comes to older adults, language matters: Journal of the American Geriatrics Society adopts modified American Medical Association Style. *J. Am. Geriatr. Soc.* 65 1386–1388. 10.1111/jgs.14941 28568284

[B39] MacNeilage P. Banks M. DeAngelis G. Angelaki D. (2010). Vestibular heading discrimination and sensitivity to linear acceleration in Head and World Coordinates. *J. Neurosci.* 30 9084–9094. 10.1523/JNEUROSCI.1304-10.2010 20610742 PMC2914270

[B40] Maurer C. Peterka R. (2005). A new interpretation of spontaneous sway measures based on a simple model of human postural control. *J. Neurophysiol.* 93 189–200. 10.1152/jn.00221.2004 15331614

[B41] Merfeld D. (2011). Signal detection theory and vestibular thresholds: I. Basic theory and practical considerations. *Exp. Brain Res.* 210 389–405. 10.1007/s00221-011-2557-7 21359662 PMC3096492

[B42] Nashner L. Peters J. (1990). Dynamic posturography in the diagnosis and management of dizziness and balance disorders. *Neurol. Clin.* 8 331–349. 10.1016/S0733-8619(18)30359-1 2193215

[B43] Nashner L. Black F. Wall C. (1982). Adaptation to altered support and visual conditions during stance: Patients with vestibular deficits. *J. Neurosci.* 2 536–544. 10.1523/JNEUROSCI.02-05-00536.1982 6978930 PMC6564270

[B44] Pasma J. Engelhart D. Maier A. Schouten A. van der Kooij H. Meskers C. (2015). Changes in sensory reweighting of proprioceptive information during standing balance with age and disease. *J. Neurophysiol.* 114 3220–3233. 10.1152/jn.00414.2015 26424578 PMC4686291

[B45] Perneger T. (1998). What’s wrong with Bonferroni adjustments. *BMJ* 316 1236–1238. 10.1136/bmj.316.7139.1236 9553006 PMC1112991

[B46] Peterka R. (2002). Sensorimotor integration in human postural control. *J. Neurophysiol.* 88 1097–1118. 10.1152/jn.2002.88.3.1097 12205132

[B47] Peterka R. Statler K. Wrisley D. Horak F. (2017). Postural compensation for unilateral vestibular loss. *Front. Neurol.* 2:57. 10.3389/fneur.2011.00057 21922014 PMC3167354

[B48] Priesol A. Valko Y. Merfeld D. Lewis R. (2014). Motion perception in patients with idiopathic bilateral vestibular hypofunction. *Otolaryngol. Head Neck Surg.* 150 1040–1042. 10.1177/0194599814526557 24647642

[B49] Scharre D. Chang S. Murden R. Lamb J. Beversdorf D. Kataki M. (2010). Self-administered Gerocognitive Examination (SAGE): A brief cognitive assessment instrument for Mild Cognitive Impairment (MCI) and early dementia. *Alzheimer Dis. Assoc. Disord.* 24 64–71. 10.1097/WAD.0b013e3181b03277 20220323

[B50] Semenov Y. Bigelow R. Xue Q. Lac S. Agrawal Y. (2016). Association between vestibular and cognitive function in US adults: Data from the National Health and Nutrition Examination Survey. *GERONA* 71 243–250. 10.1093/gerona/glv069 26219850 PMC5864155

[B51] Suzuki J. Cohen B. Bender M. (1964). Compensatory eye movements induced by vertical semicircular canal stimulation. *Exp. Neurol.* 9 137–160. 10.1016/0014-4886(64)90013-5 14126123

[B52] Valko Y. Lewis R. Priesol A. Merfeld D. (2012). Vestibular labyrinth contributions to human whole-body motion discrimination. *J. Neurosci.* 32 13537–13542. 10.1523/JNEUROSCI.2157-12.2012 23015443 PMC3467969

[B53] van der Kooij H. Peterka R. (2011). Non-linear stimulus-response behavior of the human stance control system is predicted by optimization of a system with sensory and motor noise. *J. Comput. Neurosci.* 30 759–778. 10.1007/s10827-010-0291-y 21161357 PMC3108015

[B54] van Kordelaar J. Pasma J. Cenciarini M. Schouten A. van der Kooij H. Maurer C. (2018). The reliance on vestibular information during standing balance control decreases with severity of vestibular dysfunction. *Front. Neurol.* 9:371. 10.3389/fneur.2018.00371 29915556 PMC5994722

[B55] Voorhees R. (1990). Dynamic posturography findings in central nervous system disorders. *Otolaryngol. Head Neck Surg.* 103 96–101. 10.1177/019459989010300114 2117737

[B56] Wagner A. (2023). *Uncovering vestibular contributions to age-related imbalance.* Ph.D. thesis. Columbus, OH: The Ohio State University.

[B57] Wagner A. Akinsola O. Chaudhari A. Bigelow K. Merfeld D. (2021a). Measuring vestibular contributions to age-related balance impairment: A review. *Front. Neurol.* 12:635305. 10.3389/fneur.2021.635305 33633678 PMC7900546

[B58] Wagner A. Merfeld D. (2023). A modified two-dimensional sensory organization test that assesses both anteroposterior and mediolateral postural control. *Front. Rehabil. Sci.* 4:1166859. 10.3389/fresc.2023.1166859 37284337 PMC10239846

[B59] Wagner A. Chaudhari A. Merfeld D. (2021b). Might vestibular “Noise” cause subclinical balance impairment and falls? *Int. J. Phys. Med. Rehabil.* 9:001. 35211643 PMC8865383

[B60] Wagner A. Kobel M. Merfeld D. (2021c). Impact of canal-otolith integration on postural control. *Front. Integr. Neurosci.* 15:773008. 10.3389/fnint.2021.773008 34970126 PMC8713561

[B61] Wagner A. Kobel M. Merfeld D. (2022a). Impacts of rotation axis and frequency on vestibular perceptual thresholds. *Multisens. Res.* 5 1–29. 10.1163/22134808-bja10069 35065535

[B62] Wagner A. Kobel M. Tajino J. Merfeld D. (2022b). Improving self-motion perception and balance through roll tilt perceptual training. *J. Neurophysiol.* 128 619–633. 35894439 10.1152/jn.00092.2022 PMC9448335

[B63] Wolfe J. Kluender K. Dennis L. Bartoshuk L. Herz R. Lederman S. (2021). *Sensation and perception. Sixth edition.* Cary, NC: Oxford University Press.

