# Supplementary figures and images for: Increased roll tilt thresholds are associated with subclinical postural instability in asymptomatic adults aged 21 to 84 years
Source: Front Aging Neurosci. 2023 Aug 10;15:1207711. doi: 10.3389/fnagi.2023.1207711 (PMC10448770; doi:10.3389/fnagi.2023.1207711)

**
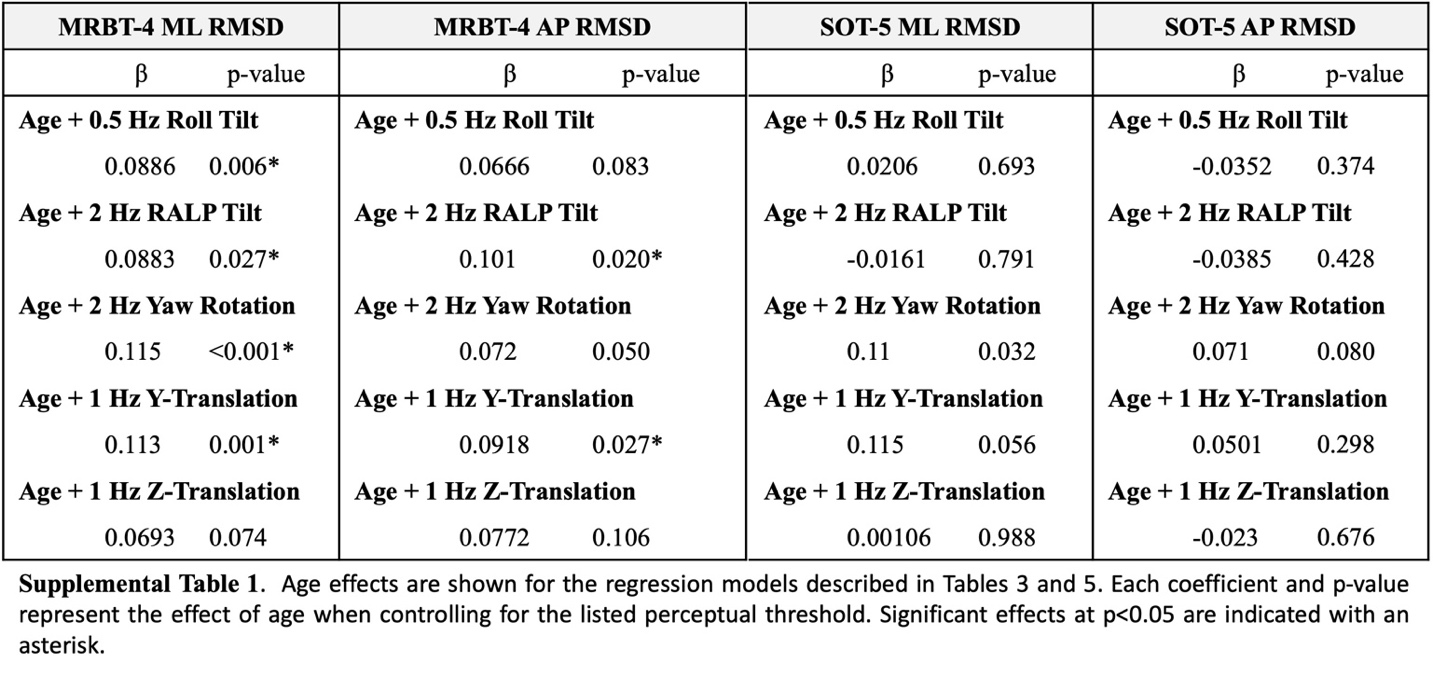
**

Supplement: Supplementary file 1 [file Table_1.DOCX]

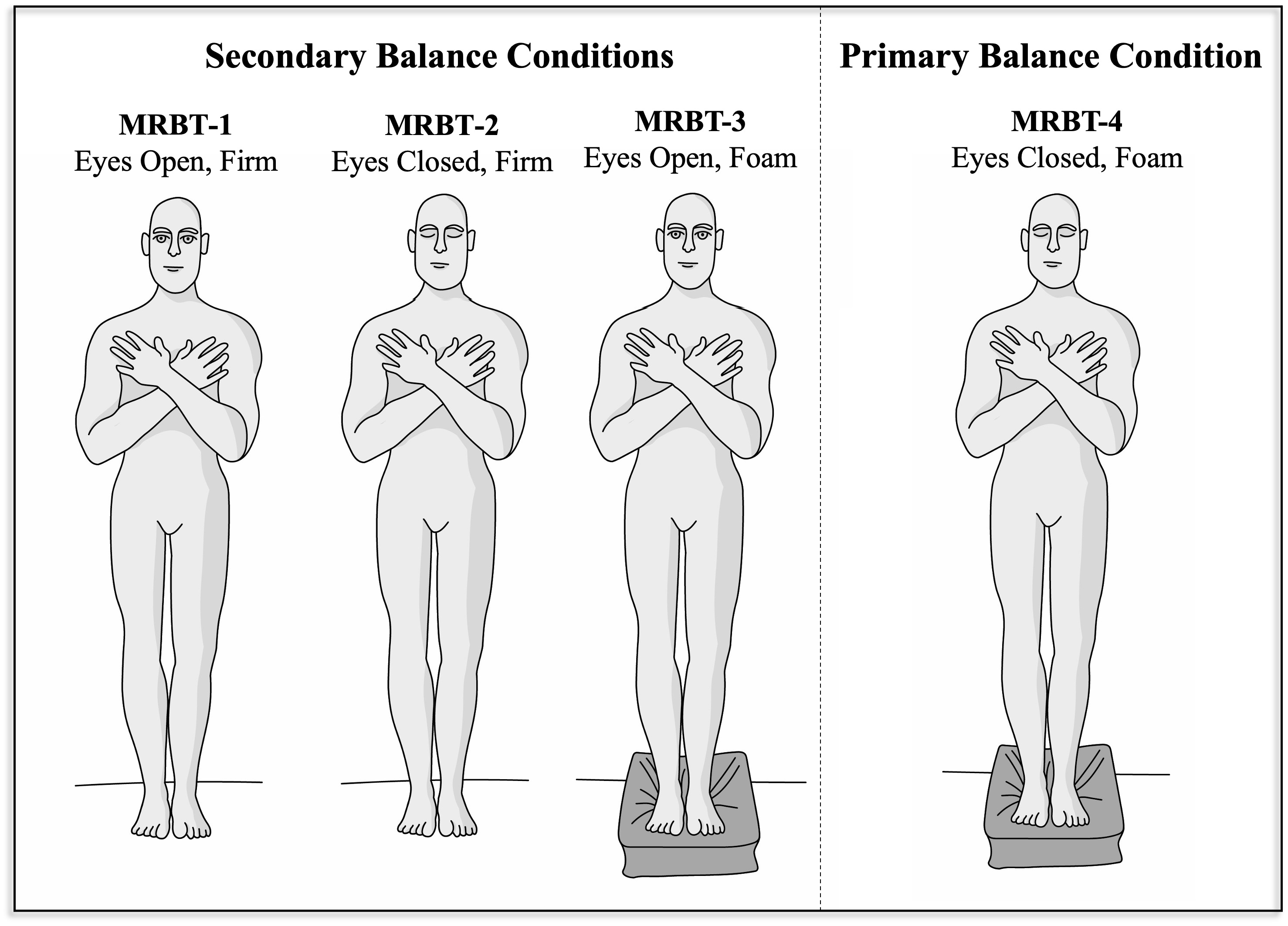

Supplement: Supplementary Figure 1 — Alternative conditions of the Modified Romberg Balance Test (MRBT) are shown. MRBT-1 involved standing on a firm surface with eyes open, MRBT-2 involved standing on a firm surface with eyes closed. MRBT-3 and MRBT-4 were performed on a compliant medium density foam pad with eyes open in MRBT-3 and closed in MRBT-4. Each condition lasted 67 s and noise cancelling headphones were worn throughout. Center of pressure data were collected at 100 Hz from a tri-axial force plate. [file Image_1.JPEG]

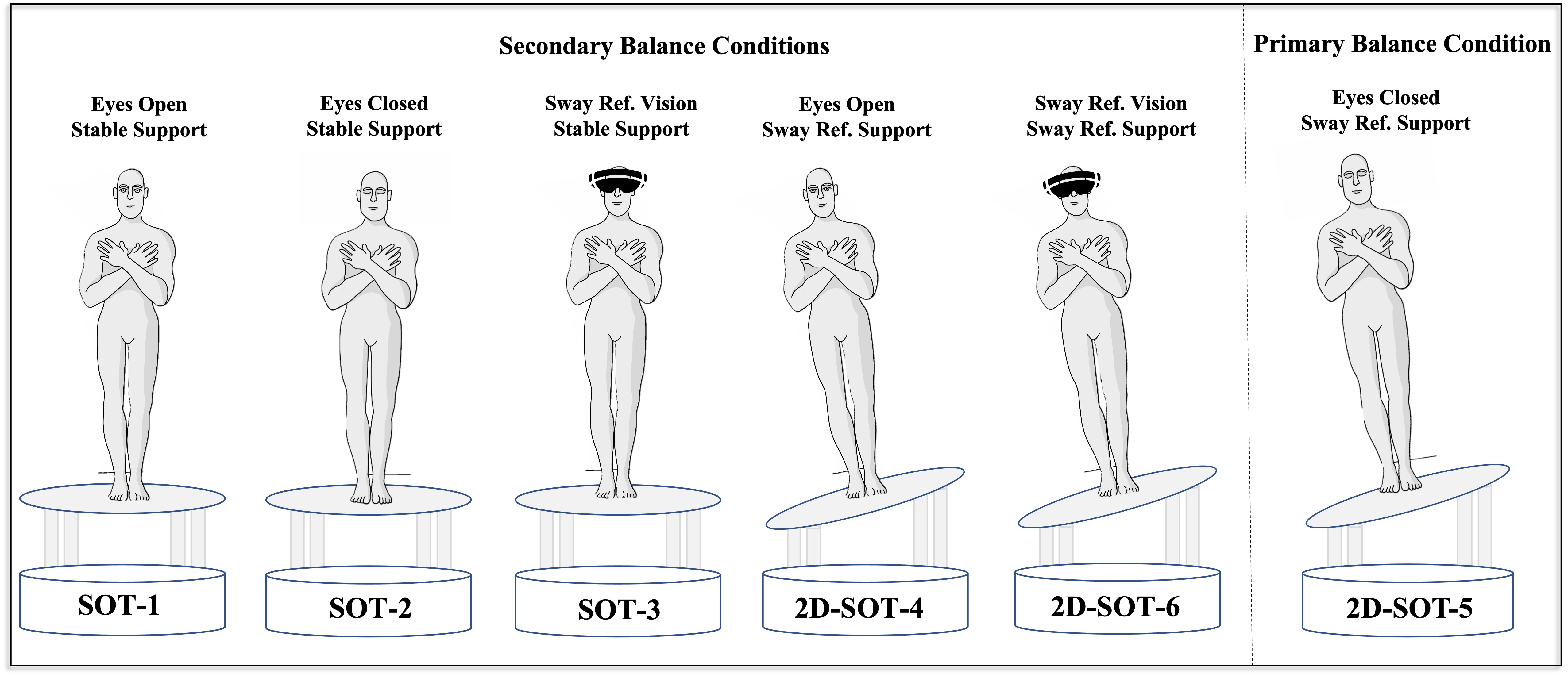

Supplement: Supplementary Figure 2 — Alternative conditions of the Sensory Organization Test (SOT) are shown. In SOT-1, SOT-2, and SOT-3 the platform was stationary whereas during 2D-SOT-4, 2D-SOT-5 (Figure 1), and 2D-SOT-6, the platform tilted in the sway referenced conditions in two dimensions (anteroposterior and mediolateral) in response to an estimate of the displacement of the center of gravity. In SOT-1 and SOT-3 the eyes were open, and the participant viewed a veridical visual scene, in SOT-2 and 2D-SOT-5 the eyes were closed, and in SOT-3 and 2D-SOT-6 the VR goggles provided a sway referenced visual scene. The black masks in SOT-3 and 2D-SOT-6 denote the use of VR to provide a sway referenced visual scene. While VR goggles were worn throughout, they are removed from the graphic in the remaining tasks to allow visualization of the eyes (open vs. closed). [file Image_2.jpg]

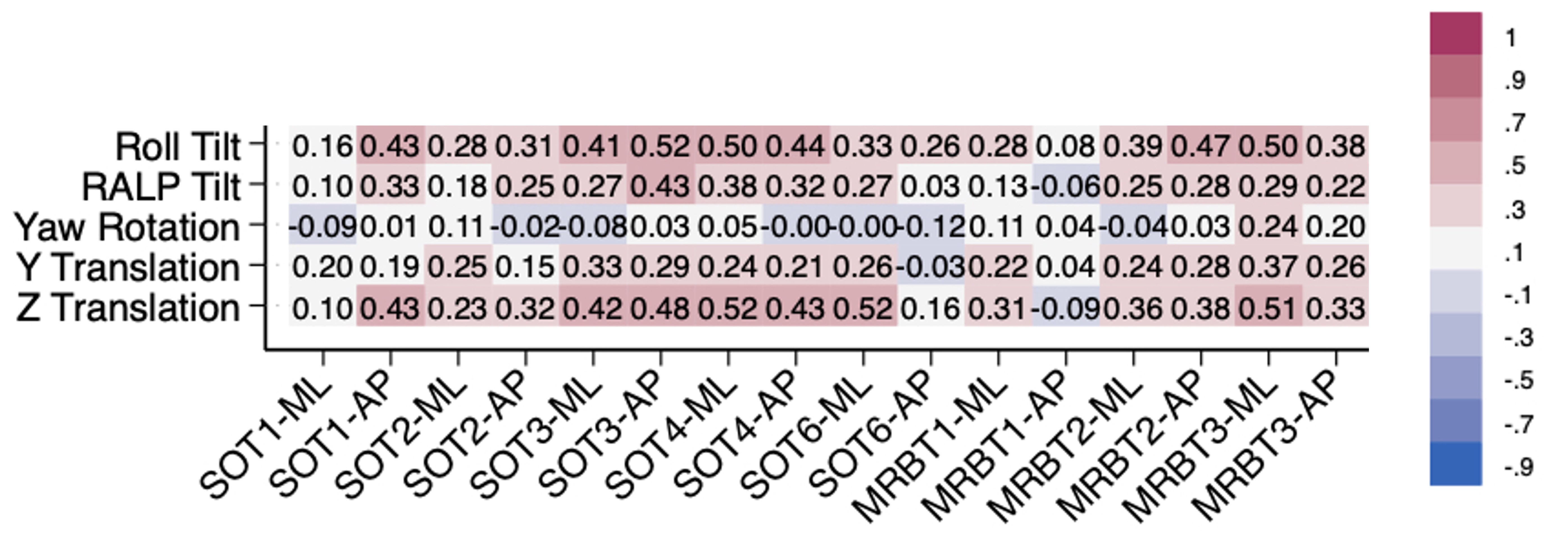

Supplement: Supplementary Figure 3 — Pearson correlation coefficients are provided within each box to demonstrate the strength of association between each of the secondary conditions of the sensory organization test (SOT conditions 1-4 and 6) and Modified Romberg Balance Test (MRBT conditions 1-3) and each of the five vestibular threshold measures—0.5 Hz roll tilt, 2 Hz RALP tilt, 2 Hz yaw rotation, 1 Hz Y-Translation, and 1 Z-Translation. The strength of the correlation is graphically indicated by the color of each cell, ranging from dark red (strongest positive association) to dark blue (strongest negative association). [file Image_3.JPEG]
